# Supplementary material for: Factors associated with knowledge and practices of COVID-19 prevention among mothers of under-2 children in Bangladesh
Source: PLOS Glob Public Health. 2024 Sep 4;4(9):e0003346. doi: 10.1371/journal.pgph.0003346 (PMC11373813; doi:10.1371/journal.pgph.0003346)
Supplement: S1 Table — (DOCX) [file pgph.0003346.s001.docx]

**S1 Table: Survey questions on knowledge related to COVID-19**

|  |  | **[1]** | **[2]** | **[3]** |
| --- | --- | --- | --- | --- |
|  |  | **Mother**  N = 2207 | **Unweighted scores** | **Weighted scores** |
|  | **Transmission, Symptoms, and Management of COVID-19** |  | **22** | **50** |
|  | **Transmission** |  |  |  |
| **K3** | **Tell me about the modes of the novel coronavirus transmission.** |  |  |  |
|  | 1. Direct transmission through droplets | 24.01 | 1 | 4.167 |
|  | 2. Indirect transmission through exposure to contaminated surface | 10.83 | 1 | 4.167 |
|  | 3. Both direct and indirect transmission modes | 48.98 | 2 | 8.335 |
|  | 4. Unaware of any of the two modes of transmission | 16.18 | 0 | 0 |
| **K4** | **Do you think that people affected by the new coronavirus always reveal symptoms?** |  |  |  |
|  | 1. No | 78.16 | 1 | 8.335 |
|  | 2. Yes | 21.84 | 0 | 0 |
|  | 3. Do not know | - | 0 | 0 |
|  |  |  |  |  |
|  | **Symptoms** |  |  |  |
| **K5** | **What are the symptoms of COVID-19? (Multiple answers)** |  |  |  |
|  | 1. Fever or chills | 87.86 | 1 | 1.5152 |
|  | 2. Cough | 80.79 | 1 | 1.5152 |
|  | 3. Fatigue | 8.97 | 1 | 1.5152 |
|  | 4. Muscle or body aches | 10.92 | 1 | 1.5152 |
|  | 5. Shortness of breath or difficulty breathing | 44.99 | 1 | 1.5152 |
|  | 6. Congestion or runny nose | 23.33 | 1 | 1.5152 |
|  | 7. Sore throat | 47.76 | 1 | 1.5152 |
|  | 8. New loss of taste or smell | 4.03 | 1 | 1.5152 |
|  | 9. Headache | 25.69 | 1 | 1.5152 |
|  | 10. Diarrhea | 2.54 | 1 | 1.5152 |
|  | 11. Red or irritated eyes | 0.09 | 1 | 1.5152 |
|  | 12. Others | 0.05 | 0 | 0 |
|  | 13. Do not know | 6.80 | 0 | 0 |
|  |  |  |  |  |
|  | **Management** |  |  |  |
| **K6** | **What are the home-remedy options for people having health conditions that mimic COVID-19? (Multiple answers)** |  |  |  |
|  | 1. Getting plenty of rest | 21.25 | 1 | 2.0833 |
|  | 2. Drinking plenty of fluids | 15.45 | 1 | 2.0833 |
|  | 3. Staying home | 54.96 | 1 | 2.0833 |
|  | 4. Staying home for at least one week or until recovery | 24.74 | 1 | 2.0833 |
|  | 5. Avoiding any strenuous activity whilst unwell | 5.57 | 1 | 2.0833 |
|  | 6. Consulting a physician or health-care service | 42.86 | 1 | 2.0833 |
|  | 7. Taking cough suppressants | 18.26 | 1 | 2.0833 |
|  | 8. Taking paracetamol | 6.80 | 1 | 2.0833 |
|  | 9. Others | 0.23 | 0 | 0 |
|  | 10. Do not know | 18.03 | 0 | 0 |
|  |  |  |  |  |
|  | **Prevention of COVID-19** |  | **27** | **50** |
| **K8** | **Describe (and demonstrate) the proper hand washing technique. (Multiple answers)** |  |  |  |
|  | 1. Wet hands with water | 60.58 | 1 | 1.25 |
|  | 2. Apply enough soap to cover all hand surfaces | 84.28 | 1 | 1.25 |
|  | 3. Rub hands palm to palm | 64.20 | 1 | 1.25 |
|  | 4. Right palm over left dorsum with interlaced fingers and vice versa | 35.48 | 1 | 1.25 |
|  | 5. Palm to palm with fingers interlaced | 31.58 | 1 | 1.25 |
|  | 6. Backs of fingers to opposing palms  with fingers interlocked | 20.57 | 1 | 1.25 |
|  | 7. Rotational rubbing of left thumb clasped in right palm and vice versa | 14.59 | 1 | 1.25 |
|  | 8. Rotational rubbing, backwards and forwards with clasped fingers of right hand in left palm and vice versa | 11.06 | 1 | 1.25 |
|  | 9. Rinsing both hands with water | 41.14 | 1 | 1.25 |
|  | 10. Scrub hands for at least 20 seconds | 29.95 | 1 | 1.25 |
|  | 11. Others | - | 0 | 0 |
|  | 12. Do not know | 7.84 | 0 | 0 |
|  | 13. Refused to answer | 0.14 | 0 | 0 |
| **K9** | **What are the criteria for proper mask-wearing? (Multiple answers)** |  |  |  |
|  | 1. Verify which side is the top – this is usually where the metal strip is | 47.39 | 1 | 1.786 |
|  | 2. Identify the inside of the mask, which is usually the white side | 30.58 | 1 | 1.786 |
|  | 3. Place the mask on your face covering your nose, mouth and chin, making sure that there are no gaps between your face and the mask | 57.0 | 1 | 1.786 |
|  | 4. Pinch the metal strip so it moulds to the shape of your nose | 22.20 | 1 | 1.786 |
|  | 5. Avoid touching the mask before cleaning hands | 5.12 | 1 | 1.786 |
|  | 6. Not sharing your mask with others | 22.16 | 1 | 1.786 |
|  | 7. Cleaning hands before putting a mask on; also before and after taking it off | 3.22 | 1 | 1.786 |
|  | 8. Others | 0.14 | 0 | 0 |
|  | 9. Do not know | 17.58 | 0 | 0 |
| **K10** | **What do you understand by physical distancing?** |  |  |  |
|  | 1. Maintaining a distance of at least 3 feet or 1-meter from each other | 39.74 | 1 | 12.5 |
|  | 2. Others | 23.83 | 0 | 0 |
|  | 3. Do not know | 39.01 | 0 | 0 |
|  | 4. Refused to answer | 0.41 | 0 | 0 |
| **K7** | **How can you prevent the transmission of novel coronavirus? (Multiple answers)** |  |  |  |
|  | 1. Regularly and thoroughly clean your hands with an alcohol-based hand rub or wash them with soap and water | 62.26 | 1 | 1.389 |
|  | 2. Maintain distance from other people | 46.67 | 1 | 1.389 |
|  | 3. Avoid touching eyes, nose and mouth | 8.02 | 1 | 1.389 |
|  | 4. Covering mouth and nose with bent elbow or tissue while coughing or sneezing | 7.66 | 1 | 1.389 |
|  | 5. Staying at home | 36.97 | 1 | 1.389 |
|  | 6. Wearing a face mask while going out | 55.55 | 1 | 1.389 |
|  | 7. Avoid the 3Cs: spaces that are closed, crowded or involve close contact | 5.85 | 1 | 1.389 |
|  | 8. Cleaning and disinfecting surfaces frequently especially those which are regularly touched, such as door handles, faucets and phone screens. | 2.85 | 1 | 1.389 |
|  | 9. If you have a fever, cough and difficulty breathing, seek medical attention immediately | 8.61 | 1 | 1.389 |
|  | 10. Others | 0.36 | 0 | 0 |
|  | 11. Do not know | 10.87 | 0 | 0 |
|  |  |  |  |  |

**Note:** (a) Columns 1 and 2 report figures in percentages.
