# Supplementary material for: Factors associated with knowledge and practices of COVID-19 prevention among mothers of under-2 children in Bangladesh
Source: PLOS Glob Public Health. 2024 Sep 4;4(9):e0003346. doi: 10.1371/journal.pgph.0003346 (PMC11373813; doi:10.1371/journal.pgph.0003346)
Supplement: S2 Table — (DOCX) [file pgph.0003346.s002.docx]

**S2 Table: Survey questions on practices related to COVID-19**

|  |  | **[1]** | **[2]** | **[3]** |
| --- | --- | --- | --- | --- |
|  |  | **Mother**  N = 2207 | **Unweighted**  **scores** | **Weighted**  **scores** |
|  | **Handwashing** |  | **13** | **33.33** |
| **P1.1** | **Do you have soap/detergent at your home for your use?** |  |  |  |
|  | 1. Yes | 99.37 | 1 | 8.333 |
|  | 2. No | 0.63 | 0 |  |
| **P1.2** | **Do you have hand sanitizer/rub at your home for your use?** |  |  |  |
|  | 1. Yes | 17.85 | 1 | 8.333 |
|  | 2. No | 82.15 | 0 |  |
| **P2** | **In what cases, do you clean your hands using soap and water to prevent novel coronavirus transmission? (Multiple answers)** |  |  |  |
|  | 1. Before touching eyes, nose, and mouth | 9.65 | 1 | 0.833 |
|  | 2. Before preparing food | 59.13 | 1 | 0.833 |
|  | 3. Before eating | 87.86 | 1 | 0.833 |
|  | 4. After touching a surface or object | 3.99 | 1 | 0.833 |
|  | 5. After blowing nose, coughing, or sneezing into hands | 10.83 | 1 | 0.833 |
|  | 6. Before and after caring for someone who is sick | 5.57 | 1 | 0.833 |
|  | 7. After using the toilet | 80.11 | 1 | 0.833 |
|  | 8. After touching an animal, animal feed, or animal waste | 12.55 | 1 | 0.833 |
|  | 9. After touching garbage | 46.81 | 1 | 0.833 |
|  | 10. After coming back from outside | 46.31 | 1 | 0.833 |
|  | 10. Do not use soap at all | 0.27 | 0 | 0 |
|  | 11. Others (Specify) | - | 0 | 0 |
|  | 12. Do not know | 1.68 | 0 | 0 |
| **P3** | **What cleansing agent do you use to clean your hands to prevent the novel coronavirus transmission? (Multiple answers)** |  |  |  |
|  | 1. Only water | 1.18 | 0 | 0 |
|  | 2. Water and soap | 98.82 | 1 | 4.165 |
|  | 3. Alcohol-based hand rub/sanitizer | 6.84 | 1 | 4.165 |
|  | 4. Soil/Mud/Ash | 1.45 | 0 | 0 |
|  | 5. Others (Specify) | - | 0 | 0 |
|  | 6. Do not know | 0.32 | 0 | 0 |
|  |  |  |  |  |
|  | **Mask-wearing** |  | **4** | **33.33** |
| **P1.3** | **Do you have face masks at your home for your use?** |  |  |  |
|  | 1. Yes | 86.27 | 1 | 8.333 |
|  | 2. No | 13.73 | 0 | 0 |
| **P4** | **Do you always wear a face mask while going out of your house?** |  |  |  |
|  | 1. Always wear a face mask | 30.36 | 1 | 8.333 |
|  | 2. Sometimes wear a face mask | 41.10 | 0 | 0 |
|  | 3. Never wear a face mask | 28.55 | 0 | 0 |
| **P5** | **Do you or your HH members share their masks?** |  |  |  |
|  | 1. Yes | 97.42 | 0 | 0 |
|  | 2. No | 2.58 | 1 | 8.333 |
|  | 3. Does not have any face mask in the household | - | 0 | 0 |
| **P6** | **Does every member of your family have at least one face mask for themselves?** |  |  |  |
|  | 1. Yes | 75.62 | 1 | 8.333 |
|  | 2. No | 24.38 | 0 | 0 |
|  |  |  |  |  |
|  | **Physical distancing** |  | **2** | **33.33** |
| **P7** | **In the last 2 weeks, how many times did you join social gatherings with people other than your household members?** |  |  |  |
|  | 1. Not a single time | 60.13 | 1 | 16.667 |
|  | 2. 1 or more times | 39.87 | 0 | 0 |
| **P8** | **In the last 2 weeks, how many times do you think you stayed at least 3 feet or 1-metre away from other people?** |  |  |  |
|  | 1. Never | 48.62 | 0 | 0 |
|  | 2. Seldom | 28.86 | 0 | 0 |
|  | 3. Some of the time | 12.23 | 0 | 0 |
|  | 4. Most of the time | 10.29 | 1 | 16.667 |
|  |  |  |  |  |

**Note:** (a) Column 1 reports figures in percentages.
