# Supplementary material for: Factors associated with knowledge and practices of COVID-19 prevention among mothers of under-2 children in Bangladesh
Source: PLOS Glob Public Health. 2024 Sep 4;4(9):e0003346. doi: 10.1371/journal.pgph.0003346 (PMC11373813; doi:10.1371/journal.pgph.0003346)
Supplement: Attachment — Submitted filename: PGPH-D-24-00415_ATcomments.pdf [file pgph.0003346.s004.pdf]

PLOS Global Public Health

Factors associated with knowledge and practices of COVID-19 prevention among mothers of under-2 children in Bangladesh

--Manuscript Draft--

Manuscript Number:	PGPH-D-24-00415
Article Type:	Research Article
Full Title:	Factors associated with knowledge and practices of COVID-19 prevention among mothers of under-2 children in Bangladesh
Short Title:	Knowledge and practices of COVID-19 prevention among mothers in Bangladesh
Corresponding Author:	Zarin Tasnim, MBBS, MPH<br>BRAC University James P Grant School of Public Health<br>Dhaka, BANGLADESH
Order of Authors:	Zarin Tasnim, MBBS, MPH
	Muhammed Nazmul Islam
	Antara Roy
	Malabika Sarker
Keywords:	COVID-19 prevention; knowledge; practice; MNCH services utilization; pandemic
Abstract:	The COVID-19 pandemic extensively impacted maternal, neonatal, and child health (MNCH) in Bangladesh. Fears and misconceptions arising from a lack of knowledge related to the virus contributed to reducing the uptake of MNCH services, which eventually helped increase maternal and neonatal mortality rates during the pandemic. In this study, we assessed the knowledge and practices related to COVID-19 prevention among the mothers of under-2 children in Bangladesh. The study was conducted in May 2021 as part of a broader research project related to COVID-19 response on MNCH service utilization. We collected data from 2207 mothers in six districts of Bangladesh using a multi-stage cluster sampling technique. We constructed weighted and unweighted composite knowledge and practice scores and identified different socio-demographic characteristics associated with the scores using multilevel generalized mixed-effect linear regression models. In general, the mothers revealed poor knowledge and practices related to COVID-19. On a weighted scale of 100, the mean composite knowledge and practice scores were 32.6 (SD = 16.4) and 53.1 (SD = 13.9), respectively. The mothers presented inadequate knowledge about COVID-19 transmission, symptoms, and the recommended preventive measures. At the same time, maintaining a safe physical distance was the least practiced preventative measure (10.3%). Level of education, access to television, and internet were significantly positively associated with their knowledge and practices related to COVID-19. Knowledge score was also positively associated with the practice score (OR = 1.26; p-value <0.001). Mothers living in islands or wetlands scored poorly compared to those living in inland. The results suggest that health interventions are needed in addressing the lack of knowledge and practices related to COVID-19. Tailored educational and behavioral interventions for the mothers from lower socio-economic strata and hard-to-reach geographic locations, could effectively reduce knowledge gaps and consequently improve the uptake of MNCH services during the pandemic.
Additional Information:	
Question	Response
Financial Disclosure<br><br>Enter a financial disclosure statement that describes the sources of funding for the work included in this submission and the	The study was part of a broader project funded by the FCDO, UK. BRAC James P Grant School of Public Health was a sub-grantee of this fund and received it from BRAC HNPP to conduct the evaluation. The authors are responsible for all the findings and conclusions. These do not necessarily represent the views of the donors.

Powered by Editorial Manager® and ProduXion Manager® from Aries Systems Corporation

role the funder(s) played. This includes grants and any commercial funding of the work or authors.<br><br>This statement will be typeset if the manuscript is accepted for publication.<br><br>*Please review the [submission guidelines] and the instructions link below for detailed requirements and guidance.*	
**Competing Interests**<br><br>On behalf of all authors, disclose any competing interests that could be perceived to bias this work.<br><br>This statement will be typeset if the manuscript is accepted for publication.<br><br>*Please review the instructions link below and PLOS Global Public Health's competing interests policy to determine what information must be disclosed at submission.*	The authors have no competing interest to declare
**Data Availability**<br><br>Before publication, Authors are required to make fully available and without restriction all data underlying their findings. Please see our PLOS Data Policy page for detailed information on this policy.<br><br>A **Data Availability Statement**, detailing where the data can be accessed, is required at first submission. Insert your Data Availability Statement in the box below.<br><br>Please see the data reporting section of our submission guidelines for instructions on what you need to include in your Data	All the data associated with this manuscript have been included in the submission as supporting information file.

Availability Statement.<br><br>This statement will be typeset if the manuscript is accepted for publication.<br><br>*PLOS allows rare exemptions to address legal and ethical concerns. If you have legal or ethical restrictions, please use the box below to detail these in full sentences for the Journal team to consider.*	

Powered by Editorial Manager® and ProduXion Manager® from Aries Systems Corporation

Main manuscript file Click here to access/download;Manuscript;COVID-19_Manuscript_26 February 2024.docx

Factors associated with knowledge and practices of COVID-19 prevention among mothers of under-2 children in Bangladesh

Zarin Tasnim[1*¶], Muhammed Nazmul Islam[1¶], Antara Roy[1], Malabika Sarker[1]

[1]BRAC James P Grant School of Public Health, BRAC University, 28 Mohakhali Commercial Area, Bir Uttam AK Khandakar Rd, Dhaka 1213, Bangladesh

* Corresponding author

Email: zarintasnim45@gmail.com (ZT)

Contact No: +8801316314149

¶These authors contributed equally to this work

Abstract

The COVID-19 pandemic extensively impacted maternal, neonatal, and child health (MNCH) in Bangladesh. Fears and misconceptions arising from a lack of knowledge related to the virus contributed to reducing the uptake of MNCH services, which eventually helped increase maternal and neonatal mortality rates during the pandemic. In this study, we assessed the knowledge and practices related to COVID-19 prevention among the mothers of under-2 children in Bangladesh. The study was conducted in May 2021 as part of a broader research project related to COVID-19 response on MNCH service utilization. We collected data from 2207 mothers in six districts of Bangladesh using a multi-stage cluster sampling technique. We constructed weighted and unweighted composite knowledge and practice scores and identified different socio-demographic characteristics associated with the scores using multilevel generalized mixed-effect linear regression models. In general, the mothers revealed poor knowledge and practices related to COVID-19. On a weighted scale of 100, the mean composite knowledge and practice scores were 32.6 (SD = 16.4) and 53.1 (SD = 13.9), respectively. The mothers presented inadequate knowledge about COVID-19 transmission, symptoms, and the recommended preventive measures. At the same time, maintaining a safe physical distance was the least practiced preventative measure (10.3%). Level of education, access to television, and internet were significantly positively associated with their knowledge and practices related to COVID-19. Knowledge score was also positively associated with the practice score (OR = 1.26; *p*-value <0.001). Mothers living in islands or wetlands scored poorly compared to those living in inland. The results suggest that health interventions are needed in addressing the lack of knowledge and practices related to COVID-19. Tailored educational and behavioral interventions for the mothers from lower socio-economic strata and hard-to-reach geographic locations, could

effectively reduce knowledge gaps and consequently improve the uptake of MNCH services during the pandemic.

Keywords: COVID-19 prevention, knowledge, practice, MNCH services utilization, pandemic

Introduction

Background

The Coronavirus Disease 2019 (COVID-19) pandemic has altered the course of our history by affecting more than 767 million people and claiming about 7 million lives globally [1]. Around 28% of the global cases and 22% of the overall deaths were from Asia [2]. In particular, Bangladesh accounted for more than 2 million positive cases and about 30 thousand deaths [2].

In addition to these immediate consequences, in Bangladesh, the COVID-19 pandemic was responsible for a drastic decline in the uptake of essential healthcare services, notably in the field of maternal, neonatal, and child health (MNCH) [3–5]. Antenatal care visits dropped by 67%, breastfeeding counseling decreased by 53%, and immunization uptake decreased by 66.1% [6]. Moreover, institutional deliveries and post-natal care visits also significantly declined during the pandemic [4]. These collective declines eventually increased maternal and child mortality and morbidity in Bangladesh [3,4,7].

The conditions were similar in many lower- and lower-middle-income countries since their health literacy was low [8]. The utilization of MNCH services was further worsened by misinformation that emerged on social media and spread faster than scientifically valid information [9]. During a health emergency such as the COVID-19 pandemic, women living in such resource-poor settings are considered comparatively more vulnerable and marginalized due to their limited access to information [10–12].

To curtail COVID-19-related morbidity and mortality, the World Health Organization (WHO) recommended some specific non-pharmacological interventions along with vaccination [13]. These

non-pharmacological approaches included proper mask-wearing, frequent and appropriate hand-washing with an alcohol-based solution or soap, and maintaining a safe physical distance [14]. However, appropriate adoption of these non-therapeutic behavioral interventions is greatly influenced by people's knowledge, attitude, and practice [15–18].

Several studies have been conducted on COVID-19 preventive knowledge, attitude, and practices in various target populations, such as adults, students, healthcare workers, women, and slum dwellers [19–24]. However, to ensure proper utilization of MNCH services, mothers of young children must have knowledge of and adherence to the specific COVID-19 preventive measures, which ultimately is influenced by their knowledge and practices [25,26].

In this study, we assess the COVID-19 preventive knowledge and practices among the mothers of under-2 children in Bangladesh and identify the factors associated with their knowledge and practices. The findings will help develop interventions targeted towards mothers, neonates, and children, reduce fear and stigma associated with COVID-19, and enhance the utilization of MNCH services in Bangladesh and countries with similar contexts.

Methods

Study sites, population, and sampling

During the pandemic in 2020, six districts of Bangladesh, namely Narayanganj, Kishoreganj, Sherpur, Bogura, Bagerhat, and Bhola, had high COVID-19 infection rates and low MNCH service utilization rates. BRAC initiated a community-based COVID-19 response program in these districts to improve their COVID-19 preventive practices and MNCH service utilization (Fig 1). We assessed

the effects of this program on MNCH service utilization using a repeated cross-sectional study design.

Fig 1. Study sites

Among different MNCH services, the postnatal care (PNC) service utilization within two days after delivery was particularly low in Bangladesh (i.e., 52% according to Bangladesh Demographic and Health Survey, 2017-18) [27]. Hence, the sample size was calculated using PNC service utilization as the outcome variable. The primary target population was the mothers of children aged 2 years or less. For each wave of data, the targeted sample size was 2400 mothers/households from six districts (i.e., 400 households per district). However, in this paper, we assessed the knowledge and practices related to COVID-19 among the mothers of under-2 children in these districts. We used data from a cross-sectional survey conducted from 9th May to 28th May 2021 as a follow-up for the program evaluation initiative mentioned above. This paper succeeded a similar assessment conducted using the baseline data in December 2020 that provided all the details on sample size calculation [28].

The sampled households were selected using a multi-stage cluster sampling technique where each village was considered a cluster. We selected 25 households from each of the 96 villages to survey 2400 households. First, we randomly selected three sub-districts from each of the six districts. Then, again, we randomly selected four unions from each sub-district. Using the population-proportion-to-size (PPS) technique we calculated the required number of villages (i.e., clusters) for each union where the total number of households residing in a union was used to represent size. Then, we randomly selected the villages for the unions. Finally, 25 households that had a mother of an under-2 child were chosen from a village following a systematic random sampling approach.

This paper used data from 2207 mothers after excluding incomplete surveys and missing values. A team of 60 trained surveyors collected the data using SurveyCTO version 2.70. To cross-check the data, field supervisors revisited 5% of households during the data collection.

Variables and their measurements

To assess knowledge and practices related to COVID-19 prevention, we prepared a questionnaire based on internationally recommended guidelines for COVID-19 [14]. We asked eight questions to assess a participant's knowledge related to the transmission, symptoms, management, and prevention of COVID-19. The questions were both single-select and multiple-select in nature and had 49 correct options in total (see S1 Table). We assessed a participant's handwashing, mask-wearing, and physical distancing practices using 10 questions containing 20 correct options (see S2 Table). We counted the correct responses to construct unweighted knowledge and practice scores. However, since the number of questions as well as their correct responses varied for different domains of knowledge and practice, we balanced the scores by weighing the domains equally. The weighted knowledge and practice scores can be compared on a scale of 100 (see column 3 of S1 and S2 Tables).

Besides knowledge and practice, we asked questions about different demographic and socioeconomic features such as age, education, occupation, household size, and monthly household income. We also enquired about the education and occupation of the household head. To check whether the participant had access to information, we asked about their access to television, phone, or internet connection. Moreover, we asked whether their household shares the kitchen and toilet with other households.

Statistical analysis

First, we conducted a descriptive analysis of all the demographic- and socioeconomic features and specific questions on knowledge and practices related to COVID-19. Then, we descriptively analyzed the weighted and unweighted scores. We reported the mean scores and standard deviations for both aggregate knowledge and practice scores and their sub-domains. We ploted histograms of the distribution of scores and caompared them against standard normal distribution.

Finally, we identified different background characteristics associated with the scores using multilevel generalized linear mixed-effect models. We asked eight knowledge questions with 49 correct options and 10 practice questions with 20 correct options. The number of correct responses was assumed to follow a Bernoulli distribution for each question. We modeled the odds of correct responses using a logit link function. The models took the following functional form:

$$g\{E(Y_{ijk}|\boldsymbol{X}_{ijk}, c_{jk}, d_k)\} = \beta_0 + \boldsymbol{\beta_x X_{ijk}} + c_{jk} + d_k + \varepsilon_{ijk}$$

where Y_{ij} is the dependent variable (i.e., binary response for a knowledge or practice option) for a question i for an individual j residing in village k; $\boldsymbol{X_{ij}}$ represents the corresponding vector of observable background features such as age, education, occupation, household size, monthly household income, etc.; c_j and d_k are the random intercepts for individual- and village-level, respectively; and ε_{ij} is the random disturbance. The models used a link function, $g\{.\}$ which was assumed to follow a Bernoulli distribution. All the random intercepts were assumed to be normally distributed. We reported the adjusted Odds ratios with corresponding P-values and 95% confidence intervals. All the analyses were carried out in Stata® version 17.

Ethical considerations

The ethical review committee of the BRAC James P Grant School of Public Health (JPGSPH) at BRAC University, Bangladesh, approved the study protocol. The reference number is IRB-20-November'20-049. Before starting any data collection activity, authorizations were collected from the respective local administrative bodies of the selected sub-districts. Informed written consent was also obtained from the participants of this study. All procedures performed in this study involving human participants were in accordance with the ethical standards of JPGSPH, BRAC University, and with the 1964 Helsinki Declaration and its later amendments or comparable ethical standards. Moreover, since the survey was conducted during the COVID-19 pandemic, all the recommended protective measures (e.g., wearing face masks, and maintaining a physical distance of at least 2 meters) were taken while interviewing the participants.

Results

Socio-demographic characteristics

Table 1 demonstrates the sociodemographic profile of the respondents. The mean age of the mothers was approximately 25 years (SD 5.5, Range 16-46 years). A big chunk of the respondents were housewives (94.15%). More than half of the mothers accomplished secondary education (52.4%), however, 7.7% of them did not receive any formal education. On the other hand, more than one-third (36.8%) self-reported household heads acquired primary education; only 8% obtained higher secondary (HSC) education or above. A large share of the household heads were wage workers (37.2%) and engaged in agriculture (26.4%). The average monthly household income was

15504 BDT (SD 17060). Almost all of them owned mobile phones (98.7%) and approximately half had access to television and internet.

Table 1. Sociodemographic characteristics

	Mother<br>N = 2207
Age [years; Mean (SD)]	25.2 (5.5)
**Education**	
No formal education	7.7
Primary or less	28.7
Above primary up to SSC	52.4
HSC and above	11.2
**Occupation**	
Home-maker	94.9
Work outside	5.1
**Head's education**	
No formal education	26.2
Primary or less	36.8
Above primary up to SSC	29.0
HSC and above	8.0
**Head's occupation**	
Wage-worker	37.2
Agriculture	26.4
Service-holder	12.6
Self-employed	18.5
Home-maker	1.6
Others	3.7
Household size [members, Mean (SD)]	5.3 (1.8)
Household income [BDT., Mean (SD)]	15,504 (17,060)
= 1 if has access to television	50.3
= 1 if has access to mobile phone	98.7
= 1 if has access to internet	50.8
= 1 if shares kitchen with others	18.5
= 1 if shares toilet with others	35.8
**District**	
Bhola	12.4
Narayanganj	13.8
Bagerhat	12.7
Bogura	29.5
Kishoreganj	21.3
Sherpur	10.3

Note: (a) Unless specified otherwise, all the values are reported in percentages; (b) standard deviation of the scores are reported in parentheses.

COVID-19 preventive knowledge

For COVID-19 transmission, although nearly half of the respondents (48.98%) knew about both direct and indirect transmission, 16.18% of them had no knowledge of either. More than one-fifth of the mothers (21.84%) thought the individual affected by COVID-19 would always reveal symptoms. The most frequently reported COVID-19 symptoms by the respondents were fever or chills (87.86%), cough (80.79%), sore throat (47.76%), shortness of breath (44.99%), and headache (25.69%). A notable proportion of the respondents indicated staying at home (54.96%) and consulting a health care provider (42.86%) as the home remedy options for the symptoms that mimicked COVID-19. As preventative measures against COVID-19, nearly two-thirds of respondents indicated regular hand-washing with an appropriate solution (62.26%), and half of them indicated wearing a face mask outside the home (55.55%) and maintaining distance from others (46.67%). Additionally, the mothers were questioned regarding the proper techniques for wearing a mask, washing hands, and maintaining physical distance (S1 Table).

Approximately, 43% of the participants were not aware of covering their nose, mouth, and chin while wearing the mask. In addition, a vast majority of them (82.42%) did not have any idea regarding not sharing their masks with other people. Overall, 84.28% mothers had knowledge of using enough soap while washing hands and only 29.95% recalled the information to scrub hands for at least 20 seconds. Furthermore, more than one-third of them were aware of staying at minimum one-meter distance from others (39.74%) to prevent COVID-19. However, 17.58% did not have any idea about proper mask-wearing techniques, 7.84% on hand-washing techniques, and 39.74% on the concept of physical distancing. The correct responses to the survey questions on COVID-19 knowledge have been reported in S2 Table.

COVID-19 preventive practice

Almost all the participants had soap (99.37%) at their home. However, most of them did not clean their hands before touching eyes, nose, and mouth (90.35%). Although majority of the participants had masks at their homes (86.27%), a large number of them shared their masks with their family members (97.42%). Around 30% respondents always used a face mask while going out of their house, nonetheless, 28.55% never used any face mask. On the other hand, more than one-third of the mothers joined social gatherings (39.87%) in the past two weeks and 48.62% did not keep a safe physical distance from others. The correct responses to the survey questions on COVID-19 preventive practices have been illustrated in S2 Table.

Knowledge and practice scores

The distribution of weighted and unweighted knowledge and practice scores has been illustrated in Figs 2 and 3. The overall mean weighted knowledge score was 32.6 (SD 16.4) on a scale of 100. The distribution of standardized knowledge scores was found to be approximately normal (Figs 2A and 2B). Almost 65.93% mothers scored within the range of -1 SD and +1 SD (between the score of 16.2 and 49) and 97.28% within -2 SD to +2 SD on the COVID-19 preventive knowledge. The findings also indicated poor knowledge of COVID-19 transmission, symptoms, and management (mean score 16.4, SD 8.3) and COVID-19 prevention (mean score 16.2, SD 10.1) (Table 2).

Fig 2A. Distributions of unweighted knowledge scores

Fig 2B. Distributions of weighted knowledge scores

Table 2. Unweighted and weighted scores

	[1]	[2]	[3]	[2]	[3]
	No. of Questions	Unweighted		Weighted	
		Scale	Score	Scale	Score
**Knowledge related to COVID-19**					
Transmission, symptoms, and management	4	0 – 22	6.8 (3.2)	0 – 50	16.4 (8.3)
Prevention	4	0 – 27	8.3 (4.5)	0 – 50	16.2 (10.1)
Overall knowledge score	8	0 – 49	7.1 (15.1)	0 – 100	32.6 (16.4)
**Practices related to COVID-19**					
Handwashing	4	0 – 14	5.9 (1.7)	0 – 33.3	17.2 (4.3)
Mask-wearing	4	0 – 4	2.9 (0.9)	0 – 33.3	24.1 (7.3)
Physical distancing	2	0 – 2	0.7 (0.6)	0 – 33.3	11.7 (9.8)
Overall practice score	10	0 – 20	9.5 (2.2)	0 – 100	53.1 (13.9)

Note: (a) Unless specified otherwise, mean knowledge- and practice-scores are reported; (b) standard deviation of the scores are reported in parentheses.

On the contrary, the practice score was defined by three domains: mask-wearing, hand-washing, and physical distancing. The overall mean weighted practice score of the mothers was 53.1 (SD 13.9) on a scale of 100 (Table 2). The distribution of standard weighted practice scores approximated the normal distribution. About 76.89% mothers obtained a practice score ranging between -1 SD and +1 SD (between a score of 39.2 and 67) and 93.97% between -2 SD and +2 SD (Figs 3A and 3B). The respondents scored lowest for physical distancing practice (mean 11.7, SD 9.8) followed by hand-washing and mask-wearing. The unweighted knowledge and practice scores demonstrated similar characteristics (Figs 2 and 3).

Fig 3A. Distributions of unweighted practice scores

Fig 3B. Distributions of weighted practice scores

Factors affecting COVID-19 preventive knowledge and practice

The log-odds of correct responses and the corresponding estimates of the association between the mothers' background information and COVID-19 preventive knowledge and practice are presented in Table 3. In the knowledge domain, the odds of correct response for COVID-19-related knowledge increased as the mothers' educational qualification rose. Compared to the mothers with no formal education, the odds of correct response for COVID-19 knowledge increased significantly by 38% (CI: 1.24 – 1.54, p< .001), 58% (CI: 1.41 – 1.76, p< .001), and 111% (CI: 1.84 – 2.41, p< .001) for those with primary education, secondary education, and higher secondary education or above respectively. Likewise, the household heads' educational status had significant association with the mothers' knowledge level. On the other hand, service-holder household heads had 13% (CI: 1.03 – 1.23, p= .008) higher odds of delivering correct answers for COVID-19 knowledge in comparison to the wage-worker household heads. Mothers' knowledge score was also predicted by their access to television and internet. A significant 19% (CI: 1.13 – 1.26, p< .001) and 8% (CI: 1.02 – 1.14, p= .006) increase in odds of giving the right information on COVID-19 knowledge were found among those with access to television and internet respectively as opposed to those who did not have access. Respondents from Bogra, Bagerhat, and Sherpur had 25% (CI: 1.02 – 1.52, p= .030), 38% (CI: 1.10 – 1.75, p= .006), 53% (CI: 1.19 – 1.94, p= .001) higher odds of offering accurate answers respectively on COVID-19 knowledge compared to Bhola. We found no significant association of mothers' knowledge with their age, occupation, household size, household income, and mobile phone access. In addition, the odds of the right answer regarding the knowledge of COVID-19 preventive measures is 4% higher than the odds of correct answer on transmission, symptoms, and management which was statistically significant (Table 3).

Table 3. Regression results

	[1]	[2]
	**Knowledge**	**Practice**
**Knowledge domain**		
Transmission, symptoms, and management	**Base**	
Prevention	1.04 (0.011)	
	(1.01 – 1.06)	
**Practice domain**		
Handwashing		**Base**
Mask-wearing		3.76 (<0.001)
		(3.57 – 3.96)
Physical-distancing		0.75 (<0.001)
		(0.70 – 0.80)
Age (in years)	1.00 (0.166)	1.00 (0.462)
	(0.99 – 1.00)	(0.99 – 1.00)
**Education**		
No formal education	**Base**	**Base**
Primary or less	1.38 (<0.001)	1.09 (0.048)
	(1.24 – 1.54)	(1.00 – 1.88)
Above primary up to SSC	1.58 (<0.001)	1.09 (0.059)
	(1.41 – 1.76)	(1.00 – 1.88)
HSC and above	2.11 (<0.001)	1.17 (0.006)
	(1.84 – 2.41)	(1.05 – 1.31)
**Occupation**		
Homemaker	**Base**	**Base**
Work outside	1.06 (0.317)	0.95 (0.291)
	(0.94 – 1.20)	(0.86 – 1.05)
**Head's education**		
No formal education	**Base**	**Base**

	[1]	[2]
	**Knowledge**	**Practice**
Primary or less	1.11 (0.002)	1.04 (0.119)
	(1.04 – 1.18)	(0.99 – 1.10)
Above primary up to SSC	1.21 (<0.001)	1.06 (0.059)
	(1.13 – 1.30)	(1.00 – 1.12)
HSC and above	1.19 (0.003)	1.12 (0.022)
	(1.06 – 1.34)	(1.02 – 1.23)
**Head's occupation**		
Wage-worker	**Base**	**Base**
Agriculture	0.99 (0.737)	1.01 (0.855)
	(0.93 – 1.06)	(0.95 – 1.06)
Service-holder	1.13 (0.008)	1.05 (0.20)
	(1.03 – 1.23)	(0.98 – 1.13)
Self-employed	1.07 (0.061)	1.06 (0.069)
	(1.00 – 1.15)	(1.00 – 1.12)
Home-maker	0.90 (0.294)	0.99 (0.881)
	(0.74 – 1.10)	(0.84 – 1.16)
Others	1.02 (0.825)	1.07 (0.259)
	(0.89 – 1.16)	(0.95 – 1.19)
Household size (no. of members)	1.00 (0.851)	1.01 (0.182)
	(0.98 – 1.01)	(1.00 – 1.02)
Household income (standardized)	1.00 (0.382)	1.00 (0.554)
	(0.99 – 1.00)	(0.99 – 1.00)
= 1 if has access to television	1.19 (<0.001)	1.01 (0.575)
	(1.13 – 1.26)	(0.98 – 1.06)
= 1 if has access to mobile phone	0.97 (0.771)	1.15 (0.142)
	(0.78 – 1.21)	(0.96 – 1.37)
= 1 if has access to internet	1.08 (0.006)	0.99 (0.609)

	[1]	[2]
	**Knowledge**	**Practice**
	(1.02 – 1.14)	(0.95 – 1.03)
= 1 if shares kitchen with others	0.97 (0.357)	1.03 (0.275)
	(0.90 – 1.04)	(0.97 – 1.10)
= 1 if shares toilet with others	0.92 (0.012)	0.96 (0.076)
	(0.87 – 0.98)	(0.91 – 1.00)
**District**		
Bhola	**Base**	**Base**
Narayanganj	1.15 (0.225)	1.12 (0.032)
	(0.92 – 1.45)	(1.01 – 1.25)
Bagerhat	1.38 (0.006)	1.18 (0.002)
	(1.10 – 1.75)	(1.06 – 1.31)
Bogura	1.25 (0.030)	1.16 (0.001)
	(1.02 – 1.52)	(1.06 – 1.28)
Kishoreganj	0.81 (0.055)	0.99 (0.754)
	(0.66 – 1.00)	(0.90 – 1.08)
Sherpur	1.53 (0.001)	1.24 (<0.001)
	(1.19 – 1.94)	(1.11 – 1.38)
Knowledge score (standardized)		1.26 (<0.001)
		(1.23 – 1.29)
Observations	1,08,143	44,100
Wald statistics (*p*-value)	552.7 (<0.001)	3282.6 (<0.001)

Note: (a) *P*-values and 95% confidence intervals are reported in parentheses.

In contrast, for the practice domain, the odds of mask-wearing practices significantly increased by 276% (CI: 3.57 – 3.96, $p < .001$) compared to the handwashing practice. On the other hand, the odds of physical distancing decreased by 25% (CI: 0.70 – 0.80, $p < .001$) in comparison to handwashing, which was statistically significant. Mothers with a higher secondary education or above had a 17%

higher (CI: 1.05 – 1.31, *p*= .006) odds of practicing preventive measures as opposed to the mothers with no formal education. Similarly, the odds of mothers' preventive practices increased by 12% (CI: 1.02 – 1.23, *p*= .022) in the households headed by individuals with higher secondary education or above compared to the households headed by individuals with no formal education. Mothers from inlands, such as, Bogra, Bagerhat, and Sherpur had 16% (CI: 1.06 – 1.28, *p*= .001), 18% (CI: 1.06 – 1.31, *p*= .002), and 53% (CI: 1.11 – 1.38, *p*< .001) higher odds of following COVID-19 preventive precautions as opposed to wetlands or islands such as, Bhola. We found no statistically significant association between mothers' preventive practices score and their age, occupation, the occupation of the household head, household size, household income, or access to television and internet. Moreover, the mothers' COVID-19 preventive practice was significantly associated with their knowledge score. The odds of COVID-19 preventive practice was 26% greater among the mothers with knowledge of COVID-19 (Table 3).

Discussion

The present study provided evidence on the knowledge and practice level of COVID-19 preventive measures of the mothers under-2 children and illustrated the factors affecting their knowledge and practice level.

The most obvious finding to emerge from the analysis is the poor COVID-19 preventive knowledge and practice level among the respondents. The present study revealed that participants lacked comprehensive understanding of COVID-19 transmission, symptoms, and precise techniques of preventative practices appropriate mask-wearing and hand-washing guidelines, as well as a clear understanding of social distancing practice. The COVID-19-infected individual will continue to spread the virus unless he or she is at least aware of the common symptoms. Many of them did not

have the understanding of the prohibition against mask sharing; hence, practically almost all of them shared masks with others despite possessing sufficient masks for themselves. The aforementioned findings tie with similar studies conducted in Bangladesh, Pakistan, and Iran among different vulnerable population groups including slum dwellers, pregnant women, and the elderly [23,29–31] where the overall responses for knowledge level ranged between 27- 44%. If the community is unaware of the specific and detailed instructions and guidelines for preventing COVID-19, even the greatest efforts to control the pandemic may fail.

Conversely, the analysis found evidence for the substandard preventive practices of maintaining social distancing against COVID-19, followed by washing hands when necessary and wearing masks to prevent the infection. This finding of COVID-19 practice level is comparable to the findings in Ethiopia and Nigeria among farmers and sex workers, where practice levels of 28% and 41% were observed respectively [32,33]. However, the deficient knowledge and practice level in the present study is contrary to some of the prior studies which suggest satisfactory knowledge level ranged between 80-90% in China, Malaysia, and Bangladesh [34–36] and practice level 76- 93% in India, Vietnam, and China [36–38]. Furthermore, it is somewhat surprising that no change was observed between the current endline study and the previous baseline study conducted in the same districts where the intervention was implemented for a long six-month period [28].

The suboptimal level of knowledge of the participant can be attributed to their relatively lower education level (equal to or below the secondary level) and reduced exposure to information, possibly because of their limited access to television and the internet. It is obvious that inadequate and poor knowledge negatively affects preventive practices. In addition to the proper knowledge level, COVID-19 preventive practices also depend on health literacy, perceived risk of infection, individual and social motivation, positive perceptions of the interventions, and implementation of

government rules and regulations to force them following the preventive measures [36–38,39]. The aforementioned facts can explain why the respondents did not follow the preventive precautions in the current study.

The regression results demonstrated that the mothers who were aware of the transmission, symptoms, and management of COVID-19 were more likely to know about preventive measures such as mask-wearing, hand-washing, and social distancing. Again, those with a deeper understanding of COVID-19 were more likely to maintain the preventative measures. An important practical implication of this finding is to target comprehensive awareness of COVID-19 to facilitate compliance with preventive precautions of the target population while designing interventions. The above finding corroborates with previous studies conducted in Bangladesh, Ghana, and South Korea [40-42].Additionally, educated mothers were more successful in acquiring adequate knowledge about COVID-19 and complying with the preventive practices in the present study, which is broadly consistent with earlier evidence [28,42]. This finding potentially implies that individuals with comparatively lower educational status might be more vulnerable to COVID-19. On the other hand, the mothers with formal employment, and accessibility to television and internet tended to be more knowledgeable about COVID-19 compared to the other mothers in our study which is also identical to previous research [42,43]. This was probably the result of exposure to information and government regulations regarding the pandemic in a formal workplace setting. The aforementioned media may therefore be a top priority in planning long-term control strategies of the pandemic.

Furthermore, in the present study, the wetlands had significantly poor COVID-19 preventive knowledge and practice level among all other districts. This finding is hardly distinguishable from another nationwide survey in Bangladesh [21]. Possible explanations include the fact that the study

sites around wetlands were located in the char and haor regions that were hard to reach. Besides, both districts have the low literacy rate in comparison to the other districts in the present study [44]. One of the issues that emerges from the above finding is that effective pandemic control may necessitate tailored interventions for risk communication and message dissemination in regions that are geographically isolated, difficult to access, and have a lower educational status.

Limitations and strengths

One of the limitations of this study was reporting bias. The participants' self-reported data on COVID-19 preventive practices were collected through face-to-face interviews. Their reported data on COVID-19 preventive practices were not affirmed by observation or other data collection methods. In addition, the participants were asked about some COVID-19 preventive measures in the last two weeks that might create unintentional recall bias. Last but not the least, since this study was a cross-sectional study, no causal inference could be drawn.

Despite these limitations, this study used robust methodology using a face-to-face interview with a large sample size and a random sampling technique. Thus, the findings could be generalized to comparable settings.

Conclusion

In the current study, mothers of under-2 children demonstrated an overall lack of knowledge and practices related to COVID-19 prevention. There was a significant association between respondents' COVID-19-related knowledge and their level of education and access to information. Moreover, mothers' overall education and specific knowledge of COVID-19 prevention are also interlinked with their preventive practices. Limitations in comprehensive understanding of COVID-19 and its

prevention resulted in a significant drop in their preventive practices. Hence, individuals with lower levels of education and poor knowledge of COVID-19 are more vulnerable to COVID-19.

This study reinforces the importance of targeted interventions for women in resource-poor settings. Tailoring messages based on their educational status and geographical location can help remove their fear and stigma related to COVID-19 and improve their knowledge and precautionary practices. Governments and development partners should design a comprehensive community-based health education program for the mothers, with special attention to COVID-19 preventive knowledge and practices. It will eventually help maximize the uptake of essential MNCH services and reduce maternal and neonatal mortality in Bangladesh during pandemics like COVID-19.

Acknowledgment

We convey our heartfelt gratitude to all the survey participants, local administrative offices of the study areas, enumerators, data collection supervisors, and other research staff for their contribution to this study.

Author Contributions

MS and MNI designed the study. MNI, MS, and AR developed the study instruments. MNI and ZT analyzed the data. ZT and MNI drafted the manuscript. All authors provided feedback and approved the final version submitted for publication.

Data Availability Statement

All the data associated with this manuscript have been included in the submission as supporting information file.

Funding

The study was part of a broader project funded by the FCDO, UK. BRAC James P Grant School of Public Health was a sub-grantee of this fund and received it from BRAC HNPP to conduct the

evaluation. The authors are responsible for all the findings and conclusions. These do not necessarily represent the views of the donors.

Conflict of Interest

The authors have no conflict of interest to declare.

References

1. WHO. WHO Coronavirus (COVID-19) Dashboard [Internet]. 2023 [cited 2023 Jul 2]. Available from: https://covid19.who.int

2. Worldometer. COVID-19 coronavirus pandemic [Internet]. 2023 [cited 2023 Feb 4]. Available from: https://www.worldometers.info/coronavirus/

3. Ahmed T, Rahman AE, Amole TG, Galadanci H, Matjila M, Soma-Pillay P, et al. The effect of COVID-19 on maternal newborn and child health (MNCH) services in Bangladesh, Nigeria and South Africa: call for a contextualised pandemic response in LMICs. International Journal for Equity in Health [Internet]. 2021 Mar 15 [cited 2023 Jul 2];20(1):77. DOI: 10.1186/s12939-021-01414-5. Available from: https://doi.org/10.1186/s12939-021-01414-5

4. Ainul S, Hossain M, Bhuiyan M, Hossain SM, Rob U, Bajracharya A. Trends in maternal health services in Bangladesh before, during and after COVID-19 lockdowns: evidence from national routine service data. Population Council Knowledge Commons [Internet]. 2020 Oct 1; DOI: 10.31899/rh14.1037. Available from: https://knowledgecommons.popcouncil.org/departments_sbsr-rh/1299/

5. Ehsan SMA, Jahan F. Analysing the impact of COVID-19 on the mothers of Bangladesh: hearing the unheard. Journal of Public Health. 2021;30(12):2869–82. DOI: 10.1007/s10389-021-01501-5.

6. Nguyen PH, Sununtnasuk C, Pant A, Tran Mai L, Kachwaha S, Ash D, et al. Provision and utilisation of health and nutrition services during COVID-19 pandemic in urban Bangladesh. Maternal & Child Nutrition [Internet]. 2021 [cited 2021 Nov 24];17(4):e13218. DOI:

10.1111/mcn.13218. Available from: https://onlinelibrary.wiley.com/doi/abs/10.1111/mcn.13218

7. Akhter N. Maternal and neonatal deaths increase during two years of COVID-19. Prothomalo [Internet]. 2022 Sep 13 [cited 2023 Jul 2]; Available from: https://en.prothomalo.com/bangladesh/1g50fgl2yz

8. Das S, Mia MN, Hanifi SMA, Hoque S, Bhuiya A. Health literacy in a community with low levels of education: findings from Chakaria, a rural area of Bangladesh. BMC Public Health [Internet]. 2017 Feb 16 [cited 2021 Nov 24];17(1):203. DOI: 10.1186/s12889-017-4097-y. Available from: https://doi.org/10.1186/s12889-017-4097-y

9. Bhanot D, Singh T, Verma SK, Sharad S. Stigma and discrimination during COVID-19 pandemic. Frontiers in Public Health. 2020;8. DOI: 10.3389/fpubh.2020.577018.

10. Acilar A, Koca G, Karamaşa Ç. Digital divide among enterprises in a developing country. IJEBEG [Internet]. 2011 Dec 1 [cited 2023 Oct 27];3(2):1–10. Available from: https://dergipark.org.tr/en/pub/ijebeg/issue/26201/275875

11. Mantwill S, Monestel-Umaña S, Schulz PJ. The relationship between health literacy and health disparities: a systematic review. PLOS ONE [Internet]. 2015 Dec 23;10(12):e0145455. DOI: 10.1371/journal.pone.0145455. Available from: https://doi.org/10.1371/journal.pone.0145455

12. Wolf MS, Serper M, Opsasnick L, O'Conor RM, Curtis L, Benavente JY, et al. Awareness, attitudes, and actions related to COVID-19 among adults with chronic conditions at the onset of the US outbreak: a cross-sectional survey. Annals of internal medicine. 2020;173(2):100–9.

13. WHO. COVID-19 vaccines offer hope, other prevention measures must continue [Internet]. 2021 [cited 2023 Jun 30]. Available from: https://www.who.int/westernpacific/news-room/commentaries/detail-hq/covid-19-vaccines-offer-hope-but-other-prevention-measures-must-continue

14. WHO. Advice for the public on COVID-19 – World Health Organization [Internet]. 2021 [cited 2021 Nov 24]. Available from: https://www.who.int/emergencies/diseases/novel-coronavirus-2019/advice-for-public

15. Akalu Y, Ayelign B, Molla MD. Knowledge, attitude and practice towards COVID-19 among chronic disease patients at Addis Zemen Hospital, Northwest Ethiopia. Infection and Drug Resistance [Internet]. 2020 Apr 15 [cited 2023 Jul 2];13:1949–60. DOI: 10.2147/IDR.S258736. Available from: https://www.tandfonline.com/doi/abs/10.2147/IDR.S258736

16. Akintunde EA. Theories and concepts for human behavior in environmental preservation. Journal of Environmental Science and Public Health [Internet]. 2017 [cited 2023 Jul 2];1(2):120–33. DOI: 10.26502/JESPH.012. Available from: http://irepos.unijos.edu.ng/jspui/handle/123456789/2807

17. Al-Hanawi MK, Angawi K, Alshareef N, Qattan AMN, Helmy HZ, Abudawood Y, et al. Knowledge, attitude and practice toward COVID-19 among the public in the kingdom of Saudi Arabia: a cross-sectional study. Frontiers in Public Health [Internet]. 2020 [cited 2023 Jul 2];8:217. Available from: https://www.frontiersin.org/articles/10.3389/fpubh.2020.00217

18. Hines JM, Hungerford HR, Tomera AN. Analysis and synthesis of research on responsible environmental behavior: a meta-analysis. The Journal of Environmental Education [Internet].

1987 Jan 1 [cited 2023 Jul 2];18(2):1–8. DOI: 10.1080/00958964.1987.9943482. Available from: https://doi.org/10.1080/00958964.1987.9943482

19. Anwar S, Araf Y, Newaz Khan A, Ullah M, Hoque N, Sarkar B, et al. Women's knowledge, attitude, and perceptions toward COVID-19 in lower-middle-income countries: a representative cross-sectional study in Bangladesh. Frontiers in Public Health [Internet]. 2020;8:721. DOI: https://doi.org/10.3389/fpubh.2020.571689. Available from: https://www.frontiersin.org/articles/10.3389/fpubh.2020.571689/full

20. Banik R, Rahman M, Sikder MT, Rahman QM, Pranta MUR. Knowledge, attitudes, and practices related to the COVID-19 pandemic among Bangladeshi youth: a web-based cross-sectional analysis. Journal of Public Health. 2021;31(1):9–19. DOI: https://doi.org/10.1007/s10389-020-01432-7.

21. Hosen I, Pakpour AH, Sakib N, Hussain N, Al Mamun F, Mamun MA. Knowledge and preventive behaviors regarding COVID-19 in Bangladesh: A nationwide distribution. PLoS One. 2021;16(5):e0251151. DOI: https://doi.org/10.1371/journal.pone.0251151.

22. Hossain MA, Rashid MUB, Khan MAS, Sayeed S, Kader MA, Hawlader MDH. Healthcare Workers' knowledge, attitude, and practice regarding personal protective equipment for the prevention of COVID-19. Journal of Multidisciplinary Healthcare. 2021;14:229–38. DOI: https://doi.org/10.2147/JMDH.S293717.

23. Islam S, Emran GI, Rahman E, Banik R, Sikder T, Smith L, et al. Knowledge, attitudes and practices associated with the COVID-19 among slum dwellers resided in Dhaka City: a Bangladeshi interview-based survey. Journal of Public Health. 2021;43(1):13–25.

24. Kumar B, Pinky SD, Nurudden AM. Knowledge, attitude and practices towards COVID-19 guidelines among students in Bangladesh. bioRxiv. 2021;4(1):100194. DOI: https://doi.org/10.1016/j.ssaho.2021.100194.

25. Ajilore K, Atakiti I, Onyenankeya K. College students' knowledge, attitudes and adherence to public service announcements on Ebola in Nigeria: suggestions for improving future Ebola prevention education programmes. Health Education Journal. 2017;76(6):648–60.

26. Tachfouti N, Slama K, Berraho M, Nejjari C. The impact of knowledge and attitudes on adherence to tuberculosis treatment: a case-control study in a Moroccan region. Pan African Medical Journal [Internet]. 2012 [cited 2021 Nov 24];12(1). DOI: 10.4314/pamj.v12i1. Available from: https://www.ajol.info/index.php/pamj/article/view/82681

27. Niport, MoH&FW. Bangladesh demographic and health survey 2017-18 final report [Internet]. 2020 Oct [cited 2023 Jul 2]. Available from: https://dhsprogram.com/publications/publication-FR344-DHS-Final-Reports.cfm

28. Talukder A, Islam MN, Sarker M, Goswami I, Siddiqua RR, Akter F, et al. Knowledge and practices related to COVID-19 among mothers of under-2 children and adult males: a cross-sectional study in Bangladesh. BMJ Open [Internet]. 2022 May 1 [cited 2023 Feb 2];12(5):e059091. DOI: 10.1136/bmjopen-2021-059091. Available from: https://bmjopen.bmj.com/content/12/5/e059091

29. Maharlouei N, Asadi N, Bazrafshan K, Roozmeh S, Rezaianzadeh A, Zahed-Roozegar MH, et al. Knowledge and attitude regarding COVID-19 among pregnant women in Southwestern Iran in the early period of its outbreak: a cross-sectional study. The American Journal of Tropical Medicine and Hygiene. 2020;103(6):2368. DOI: 10.4269/ajtmh.20-0608.

30. Salman M, Mustafa ZU, Asif N, Shehzadi N, Hussain K, Khan TM, et al. Awareness of COVID-19 among illiterate population in Pakistan: a cross-sectional analysis. Disaster Med Public Health Prep [Internet]. 2021 [cited 2021 Dec 31];17:e31. DOI: 10.1017/dmp.2021.261. Available from: https://www.ncbi.nlm.nih.gov/pmc/articles/PMC8523976/

31. Vicerra PMM. Disparity between knowledge and practice regarding COVID-19 in Thailand: A cross-sectional study of older adults. PLoS One [Internet]. 2021 Oct 26 [cited 2021 Dec 31];16(10):e0259154. DOI: 10.1371/journal.pone.0259154. Available from: https://www.ncbi.nlm.nih.gov/pmc/articles/PMC8547694/

32. Avwioro G, Egwunyenga A, Adjekuko C, Mgbere O, Odibo E, Iyiola S, et al. Commercial sex work during novel coronavirus (SARS-CoV-2) era in the Niger Delta region: relationships between knowledge, preventive practice, and transmission potential. Int J Womens Health [Internet]. 2021 Jun 1 [cited 2021 Dec 31];13:509–23. DOI: 10.2147/IJWH.S303565. Available from: https://www.ncbi.nlm.nih.gov/pmc/articles/PMC8183186/

33. Berhanu L, Berihun G, Walle Z, Teshome D, Gizeyatu A, Abebe M, et al. COVID-19 prevention practices and associated factors among farmers in peri-urban areas of Northeastern Ethiopia. J Multidiscip Healthc [Internet]. 2021 Jul 12 [cited 2021 Dec 31];14:1843–52. DOI: 10.2147/JMDH.S321456. Available from: https://www.ncbi.nlm.nih.gov/pmc/articles/PMC8286086/

34. Azlan AA, Hamzah MR, Sern TJ, Ayub SH, Mohamad E. Public knowledge, attitudes and practices towards COVID-19: a cross-sectional study in Malaysia. PLOS ONE [Internet]. 2020 May 21 [cited 2021 Dec 31];15(5):e0233668. DOI: 10.1371/journal.pone.0233668. Available from: https://journals.plos.org/plosone/article?id=10.1371/journal.pone.0233668

35. Kundu S, Al Banna M, Sayeed A, Begum MR, Brazendale K, Hasan MT, et al. Knowledge, attitudes, and preventive practices toward the COVID-19 pandemic: an online survey among Bangladeshi residents. Journal of Public Health. 2021;31(7):1121–35. DOI: 10.1007/s10389-021-01636-5.

36. Zhong BL, Luo W, Li HM, Zhang QQ, Liu XG, Li WT, et al. Knowledge, attitudes, and practices towards COVID-19 among Chinese residents during the rapid rise period of the COVID-19 outbreak: a quick online cross-sectional survey. Int J Biol Sci [Internet]. 2020 Mar 15 [cited 2021 Dec 23];16(10):1745–52. DOI: 10.7150/ijbs.45221. Available from: https://www.ncbi.nlm.nih.gov/pmc/articles/PMC7098034/

37. Van Nhu H, Tuyet-Hanh TT, Van NTA, Linh TNQ, Tien TQ. Knowledge, attitudes, and practices of the Vietnamese as key factors in controlling COVID-19. J Community Health [Internet]. 2020 Sep 7 [cited 2021 Dec 25];45(6):1263–9. DOI: 10.1007/s10900-020-00919-4. Available from: https://www.ncbi.nlm.nih.gov/pmc/articles/PMC7476242/

38. Yousaf MA, Noreen M, Saleem T, Yousaf I. A cross-sectional survey of knowledge, attitude, and practices (KAP) toward pandemic COVID-19 among the general population of Jammu and Kashmir, India. Social Work in Public Health [Internet]. 2020 Sep 1 [cited 2021 Dec 25];35(7):569–78. DOI: 10.1080/19371918.2020.1806983. Available from: https://www.tandfonline.com/doi/full/10.1080/19371918.2020.1806983

39. Luo Y, Yao L, Zhou L, Yuan F, Zhong X. Factors influencing health behaviours during the coronavirus disease 2019 outbreak in China: an extended information-motivation-behaviour skills model. Public Health [Internet]. 2020;185:298–305. DOI:

https://doi.org/10.1016/j.puhe.2020.06.057. Available from: https://www.sciencedirect.com/science/article/pii/S0033350620302961

40. Ampofo RT, Aidoo EN. Structural equation modelling of COVID-19 knowledge and attitude as determinants of preventive practices among university students in Ghana. Scientific African [Internet]. 2022 Jul 1 [cited 2023 Jul 8];16:e01182. DOI: 10.1016/j.sciaf.2022.e01182. Available from: https://www.sciencedirect.com/science/article/pii/S2468227622000904

41. Lee M, Kang BA, You M. Knowledge, attitudes, and practices (KAP) toward COVID-19: a cross-sectional study in South Korea. BMC Public Health [Internet]. 2021 Feb 5 [cited 2023 Jul 8];21(1):295. DOI: 10.1186/s12889-021-10285-y. Available from: https://doi.org/10.1186/s12889-021-10285-y

42. Rabbani MG, Akter O, Hasan MZ, Samad N, Mahmood SS, Joarder T. COVID-19 knowledge, attitudes, and practices among people in Bangladesh: telephone-based cross-sectional survey. JMIR Form Res [Internet]. 2021 Nov 5 [cited 2021 Dec 25];5(11):e28344. DOI: 10.2196/28344. Available from: https://formative.jmir.org/2021/11/e28344

43. Rehman R, Jawed S, Ali R, Noreen K, Baig M, Baig J. COVID-19 pandemic awareness, attitudes, and practices among the Pakistani general public. Frontiers in Public Health [Internet]. 2021 [cited 2021 Dec 25];9:601. DOI: 10.3389/fpubh.2021.588537. Available from: https://www.frontiersin.org/article/10.3389/fpubh.2021.588537

44. BBS. Population and housing census [Internet]. Bangladesh Bureau of Statistics. 2023 [cited 2023 Feb 3]. Available from: https://bbs.gov.bd/site/page/47856ad0-7e1c-4aab-bd78-892733bc06eb/-

Supporting information

S1 Table. Survey questions on knowledge related to COVID-19

S2 Table. Survey questions on practices related to COVID-19

S1 Dataset. De-identified data

Click here to access/download;Figure;Fig1.tif

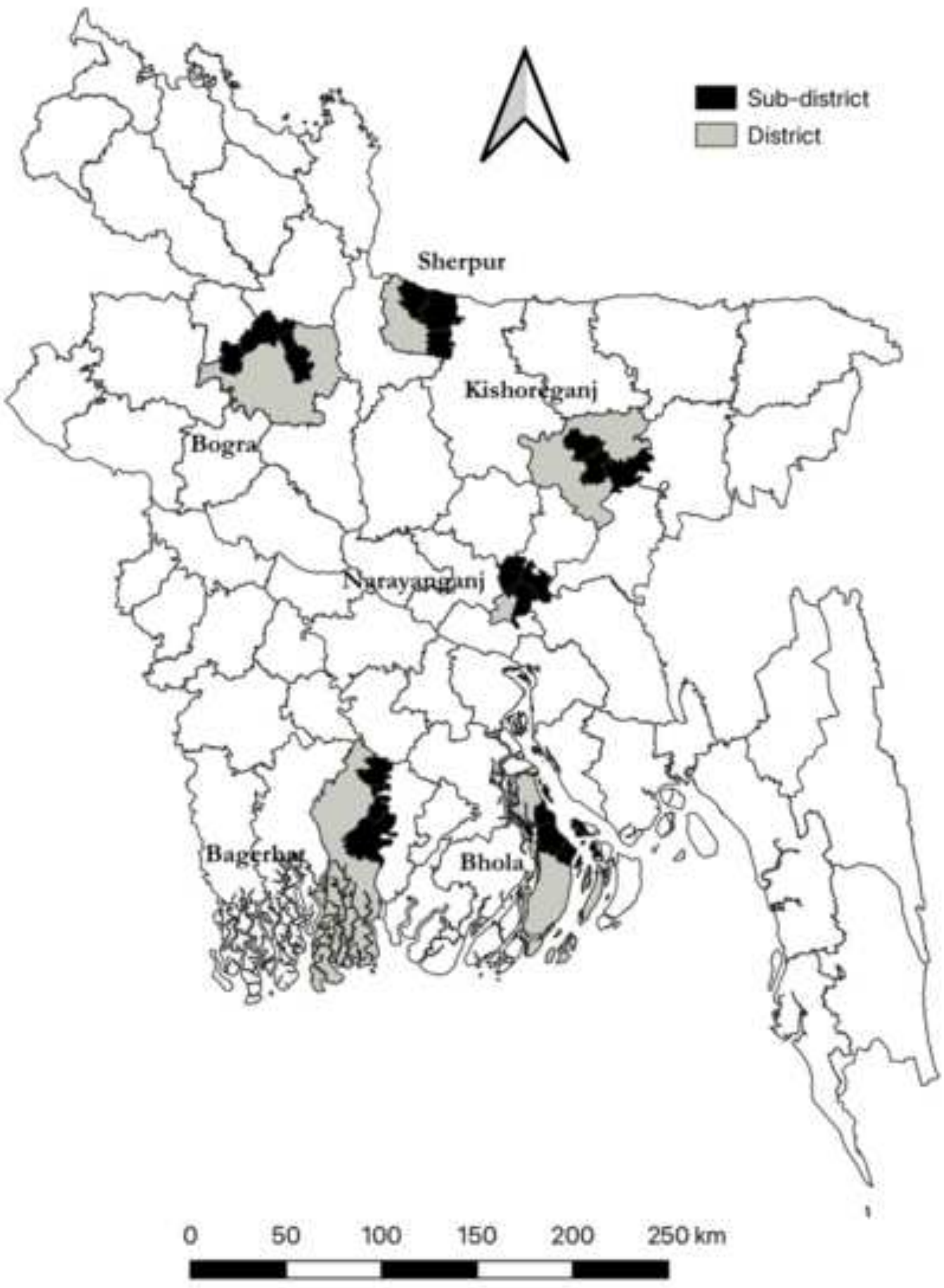

Figure 2a

Click here to access/download;Figure;Fig2a.tif

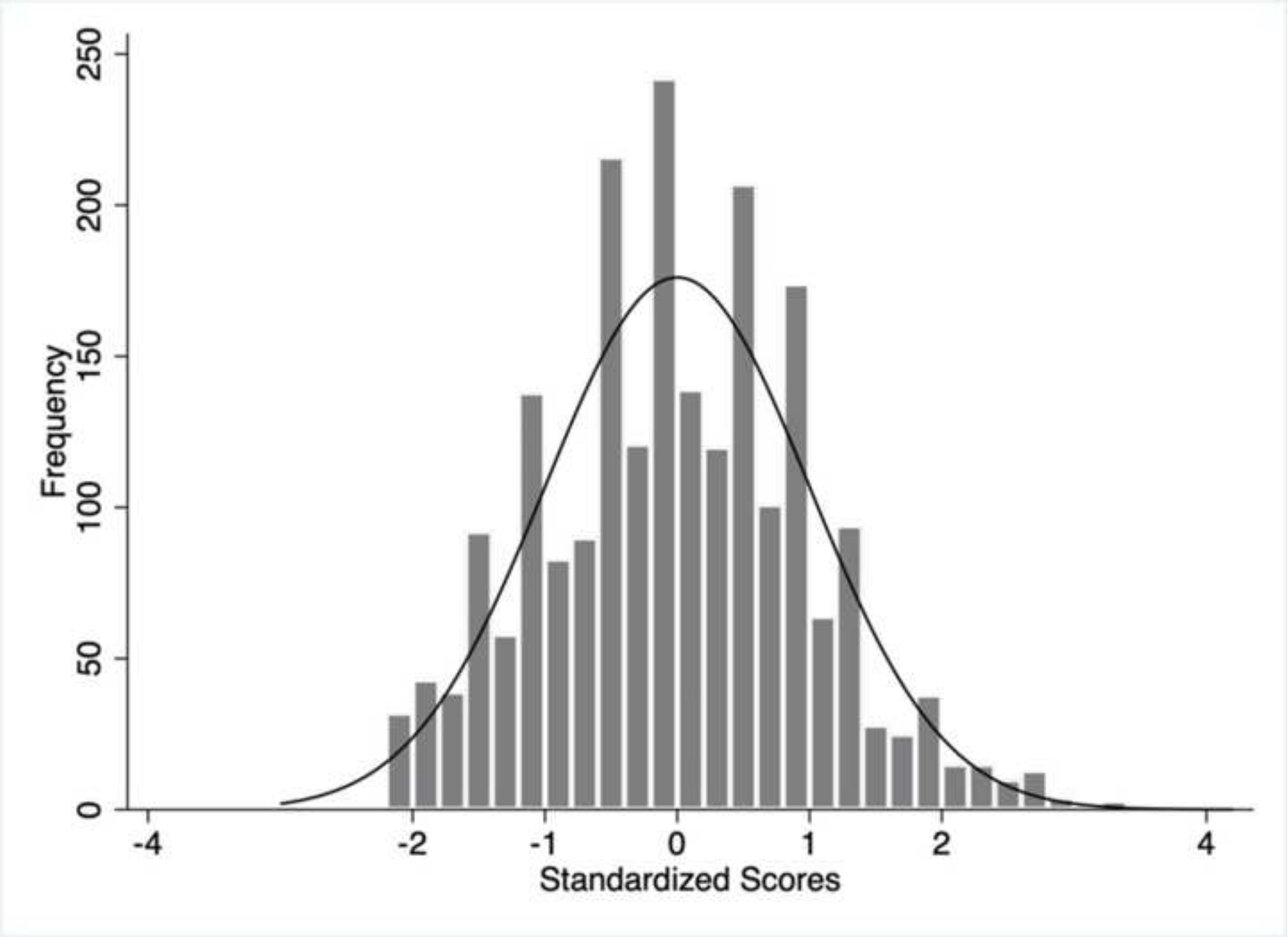

Figure 2b

Click here to access/download;Figure;Fig2b.tif

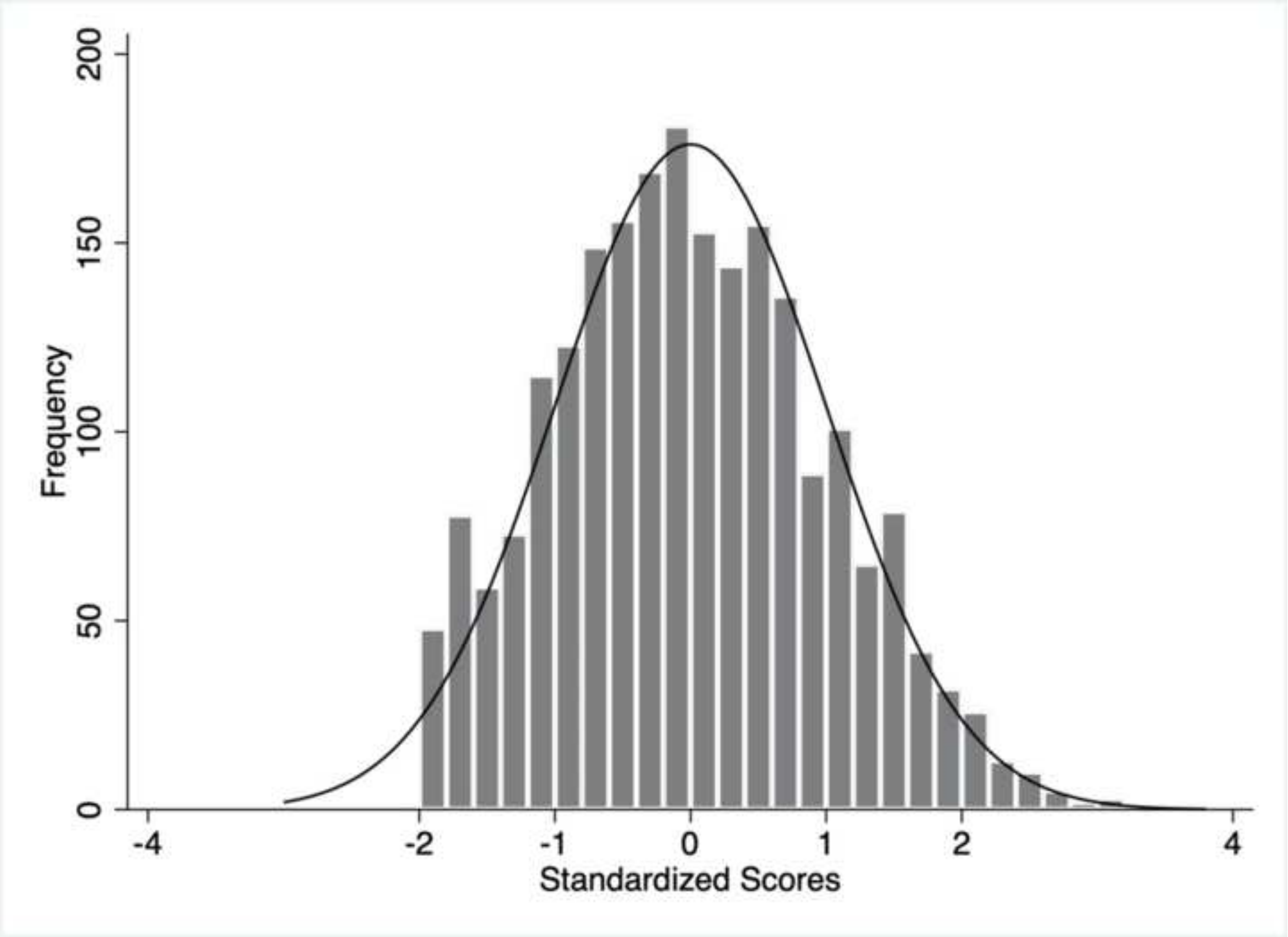

Figure 3a

Click here to access/download;Figure;Fig3a.tif

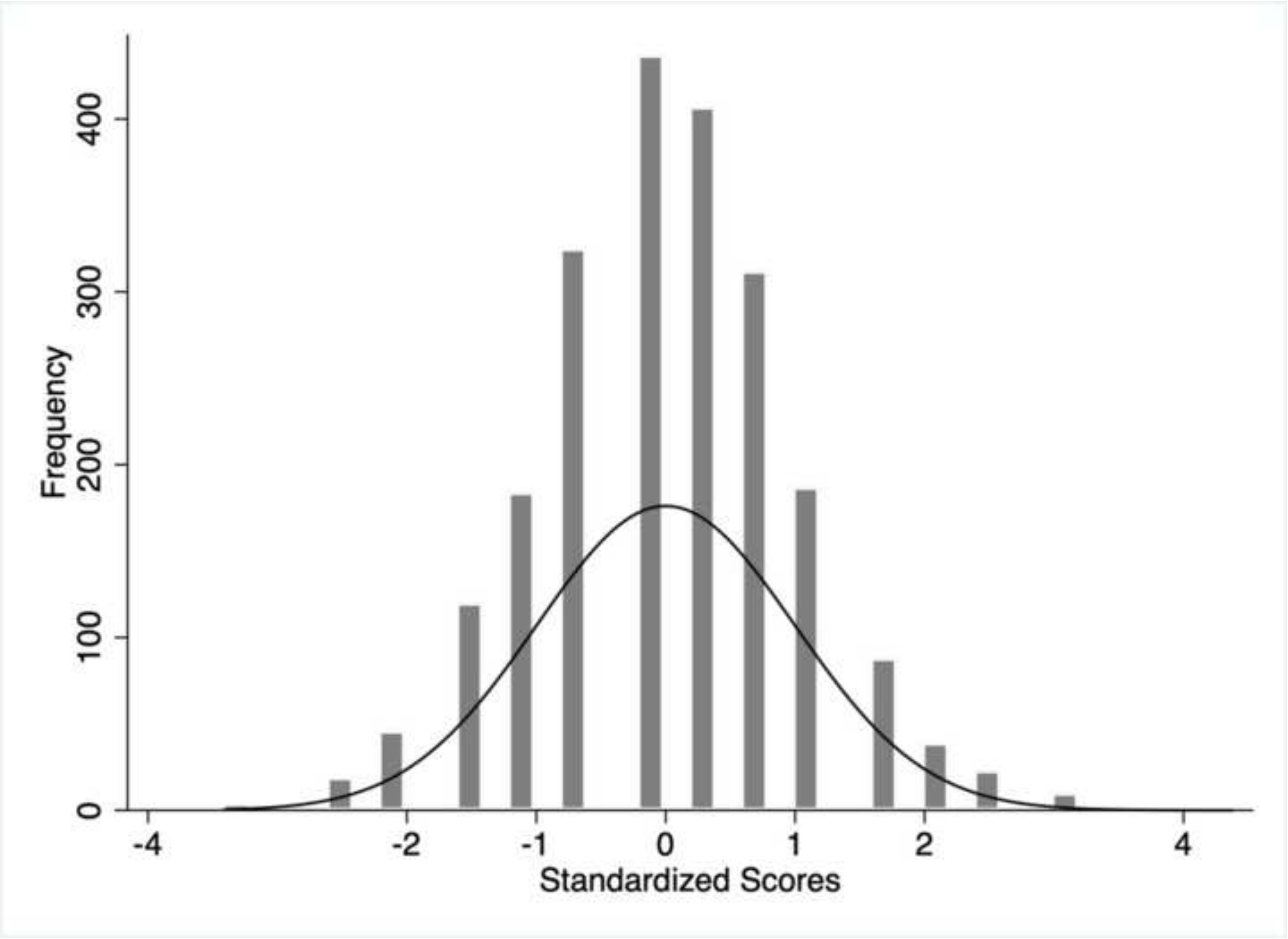

Figure 3b

Click here to access/download;Figure;Fig3b.tif

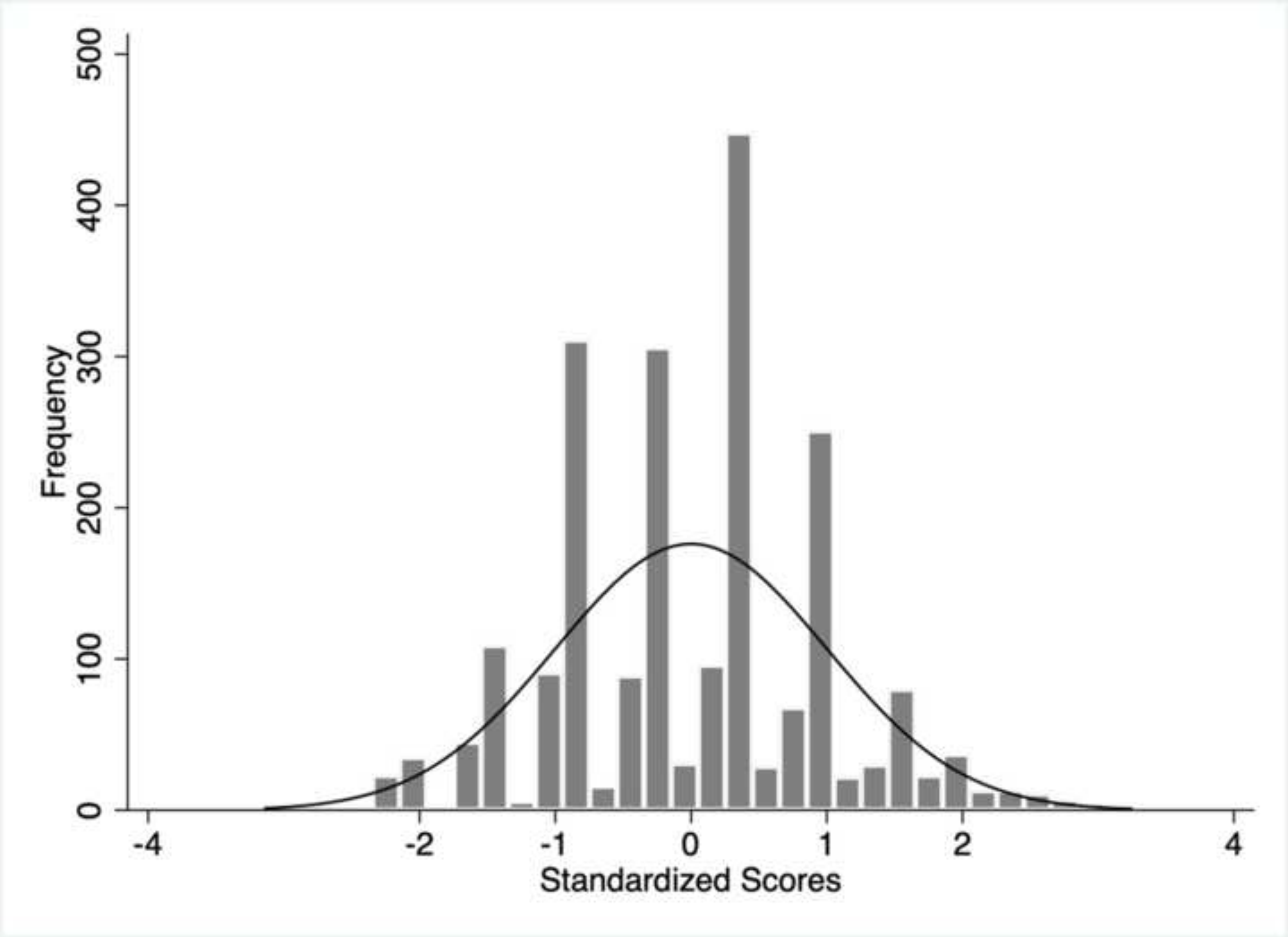

Click here to access/download
Supporting Information - Compressed/ZIP File Archive
Supporting Information.zip
