# Supplementary material for: Factors associated with knowledge and practices of COVID-19 prevention among mothers of under-2 children in Bangladesh
Source: PLOS Glob Public Health. 2024 Sep 4;4(9):e0003346. doi: 10.1371/journal.pgph.0003346 (PMC11373813; doi:10.1371/journal.pgph.0003346)
Supplement: Attachment — Submitted filename: Rebuttal letter_25 May 2024.docx [file pgph.0003346.s005.docx]

**Response to the reviewers**

**Reviewer #1**

1. The manuscript reflects methodologically sound and ethically rigorous research, with the conclusions drawn mostly aligning with the data presented. The statistical analyses seem to have been performed appropriately and rigorously. The authors have also made all data underlying the findings fully available, enabling open science practices such as replications to comparable contexts. Besides, the manuscript has been formulated in an intelligible fashion and written in standard English. Nevertheless, some constructive feedback is provided (as comments within the PDF), which are believed to further improve its methodological rigour.

**Response: We thank you for thoroughly reviewing the manuscript and providing valuable feedback and suggestions. We have carefully considered the comments and endeavored to address them with utmost care and attention to improve the clarity and overall quality of the paper.**

2. Methods: “We assessed the effects of this program on MNCH service utilization using a repeated cross-sectional study design.” This reads as a study objective (which it is not), and to clear the nuance, perhaps it can be highlighted that it was a separate endeavor and not discussed in the present report.

**Response: We have revised this writing piece to emphasize the present study's objective. (Page 5; Line number- 84)**

3. Methods: “This paper used data from 2207 mothers after excluding incomplete surveys and missing values.” The authors are advised to discuss the missingness assumption made, the implications the assumption may have on the analyses after excluding the missing observations, and how they took that into account (e.g., through any sensitivity analyses that might have been performed).

**Response: Thank you for bringing this to our attention. Initially, we gathered data from a total of 2211 mothers. However, among them, four mothers did not meet the inclusion criteria, therefore, we excluded their responses. Hence, using the term "missing value" in this instance is unsuitable. To enhance clarity, we omitted the word "missing value" from the manuscript. (Page 6; Line number 108)**

4. Methods: “Since the number of questions as well as their correct responses varied for different domains of knowledge and practice, we balanced the scores by weighing the domains equally.” The weighting method could be described or the weighting formulae could be shared for greater clarity.

**Response: We have added a description of the weighting method (Page 7; Line number 120)**

5. Methods: “We ploted histograms of the distribution of scores and caompared them against standard normal distribution.” change it to 'compared'. Might describe the purpose of this step with respect to the analyses and what strategies were taken (if any) in case the scores had a non-normal distribution (e.g., log-transformation of the outcome or using non-parametric analysis techniques).

**Response: Thank you for catching this typographical error; we have corrected it. We plotted the histograms to visualize the distribution of the scores but used individual responses in the final model. After performing the tests, we saw that the distributions of knowledge and practice scores were approximately normal. Therefore, there was no need to do any analysis for non-normal distribution. (Page- 8; Line number- 141)**

6. Methods: “Finally, we identified different background characteristics associated with the scores using multilevel generalized linear mixed-effect models.” Might describe the number of levels considered and the nature of nesting, e.g., individuals nested within households, that are nested within clusters, or something else. Might describe which effect was considered random and which was considered fixed, e.g., clustering as random effect and survey as fixed, etc. Change it to 'mixed-effects'.

**Response: We have included a sentence to explain the hierarchical structure of data, including the number of levels and the nature of nesting. (Page- 8; Line number- 147)**

7. “x𝒊𝒋 represents the corresponding vector of observable background features such as age, education, occupation, household size, monthly household income, etc.; 𝑐𝑗 and 𝑑𝑘 are the random intercepts for individual- and village-level, respectively; and 𝜀𝑖𝑗 is the random disturbance” This description can be repeated where the term 'mixed-effects model' was introduced first, for greater coherence.

**Response: Thank you for highlighting the need for more coherence. Nevertheless, we made an effort to prevent this redundancy for the readers of the paper and uphold a coherent flow of contents to the best extent possible. (Page- 9; Line number- 151)**

8. “Factors affecting COVID-19 preventive knowledge and practice” General comment on this section: Most interpretations infer causal association, whereas the nature of the modelling was predictive/prognostic at best instead of causal.

**Response: Thank you for noting this important concern regarding the interpretation of the regression results. We have modified the write-up to indicate the association instead of causal inference and made relevant revisions to the result section. (Page 14; Line number-232)**

9. “In the knowledge domain, the odds of correct response for COVID-19-related knowledge increased as the mothers’ educational qualification rose.” This reads as a casual interpretation - however, given the study design, the interpretations should only infer association. Besides, educational qualification was a categorical variable, but the interpretation suggests a linear association with the outcome.

**Response: We have updated this piece of writing. (Page 14; Line number- 235)**

10. “Compared to the mothers with no formal education, the odds of correct response for COVID-19 knowledge increased significantly by 38% (CI: 1.24 – 1.54, p< .001), 58% (CI: 1.41 – 1.76, p< .001), and 111% (CI: 1.84 – 2.41, p< .001) for those with primary education, secondary education, and higher secondary education or above respectively” Infers causal association, which is not the case.

**Response: We have rephrased accordingly. (Page 14; Line number- 236)**

11. “A significant 19% (CI: 1.13 – 1.26, p< .001) and 8% (CI: 1.02 – 1.14, p= .006) increase in odds of giving the right information on COVID-19 knowledge were found among those with access to television and internet respectively as opposed to those who did not have access.” Infers causal association, which is not the case.

**Response: We have revised accordingly. (Page 15; Line number- 244)**

12. “In contrast, for the practice domain, the odds of mask-wearing practices significantly increased by 276% (CI: 3.57 – 3.96, p< .001) compared to the handwashing practice.” (wrongly) infers causal association.

**Response: We have addressed this. (Page 18; Line number- 254)**

13. “On the other hand, the odds 245 of physical distancing decreased by 25% (CI: 0.70 – 0.80, p< .001) in comparison to handwashing, which was statistically significant.” (wrongly) infers causal association.

**Response: We have updated this. (Page 18; Line number- 255)**

14. “Similarly, the odds of mothers’ preventive practices increased by 12% (CI: 1.02 – 1.23, p= .022) in the households headed by individuals with higher secondary education or above compared to the households headed by individuals with no formal education” (wrongly) infers causal association.

**Response: We have modified this. (Page 18; Line number- 259)**

15. “The suboptimal level of knowledge of the participant can be attributed to their relatively lower education level (equal to or below the secondary level) and reduced exposure to information, possibly because of their limited access to television and the internet.” No attribution analysis was performed to draw this inference” No attribution analysis was performed to draw this inference.

**Response: Thank you for pointing this out. We have made the necessary revisions to this statement. (Page 20; Line number- 295)**

16. “One of the limitations of this study was reporting bias.” Might discuss to which direction this would bias the adjusted estimates

**Response: We have added an explanation to address this in the limitations. (Page 21; Line number- 331)**

17. “In addition, the participants were asked about some COVID-19 preventive measures in the last two weeks that might create unintentional recall bias” Might discuss how this potential measurement error could introduce residual confounding in the adjusted estimates.

**Response: We have addressed this in the limitations. (Page 21; Line number 327)**

18. “Last but not the least, since this study 326 was a cross-sectional study, no causal inference could be drawn” This is a good effort by the authors to highlight the challenges in making causal inference through such study designs. Nevertheless, an attempt was made to draw such inferences in several parts of the manuscript.

**Response: We appreciate you flagging this issue. We have addressed this in the result section. (Page 14; Line number- 232)**

19. “Despite these limitations, this study used robust methodology using a face-to-face interview with a large sample size and a random sampling technique” Might also discuss chances of unmeasured confounding and non-response bias

**Response: We have addressed this in the limitations. (Page 21; Line number 327)**

**Reviewer #2**

1. Abstract: The conclusion should be based on the assessment of the findings. The author stated that tailored educational and behavioral interventions for mothers from lower socio-economic strata and hard-to-reach geographic locations could... What was the basis for this statement since there are no such variables aligned with this statement?

**Response: Thank you for pointing this out. We concur that the terminology "lower socio-economic strata" does not reflect the findings from our study. Therefore, we substituted it with a lower maternal education level, which was found to have a significant association with both knowledge and preventive practices of COVID-19. (Page- 2; Line number- 40) However, compared to the other districts, the mothers in Bhola had lower odds of COVID-19 preventive knowledge and practices compared to other districts. The selected unions in Bhola were geographically isolated wetlands that were difficult to access. Since we found an association between the mothers’ COVID-19 preventive knowledge and practices and living in geographically remote area (Bhola), we ended up with the conclusion regarding “intervention for the mothers living in hard-to-reach geographic locations”. In the discussion section, we have also explained why mothers in geographically remote areas might need different interventions. Individuals residing in such remote geographical regions had a generally low literacy level. (Page 21; Line number- 319)**

2. Introduction/Background: In lines 45-46, use either ‘Introduction’ or ‘Background’ following the journal's instructions.

**Response: Thank you for spotting this. We have revised it accordingly. (Page 4; Line number 44)**

3. Introduction/Background: How do the study objective and findings align with fear and stigma, since these words are mentioned twice throughout the paper under background and conclusion? Since no relation to findings or discussion, what was the basis?

**Response: Thank you for mentioning this. The basis was the interlink of the mothers’ COVID-19 preventive knowledge and practice, MNCH service utilization, and fear stigma related to COVID-19. However, since the utilization of MNCH services and fear and stigma related to COVID-19 were not the prime focus of the present paper, we excluded “fear and stigma” from the manuscript.**

4. Methods: What was the procedure of the systematic random sampling approach to select the mothers from each village? Was there a minimum sample available in each selected village? In a yes or no situation, how was the sampling strategy applied? Does it vary from village to village?

**Response: We used the list of households collected from BRAC as the sampling frame. To apply the systematic random sampling, we calculated the interval (k) with the following formula:**

**Interval (k)=** $\frac{Total households with mothers under-2 child in a village (cluster)}{Number of households to be randomly drawn from each village (cluster)}$

**The first household with a mother having children under 2 was randomly selected, and the rest were chosen at kth intervals. Twenty-five households were selected from each village following systematic random sampling. The village register contained an adequate number of samples of mothers with children under 2.**

5. Results and Discussions: It is suggested to include a table as a main table with the frequency distribution of knowledge and practice questions. This may ensure a better flow to understand Table 2.

**Response: The tables containing knowledge and practice questions span almost 8 pages and encompass several domains and sub-domains related to knowledge and practice. To comply with the submission guidelines and present our findings concisely, we preferred to submit the tables as supplemental files. Nevertheless, we are willing to include the tables within the main manuscript document upon receiving the editor’s authorization and consideration.**

6. Results and Discussions: Based on the knowledge score and practice, how did the authors determine poor knowledge and practice? When can it be called good or better knowledge based on the score? What is the cutoff point for it?

**Response: The weighted knowledge and practice scores were scaled between 0 and 100. The average weighted scores for COVID-19 preventive knowledge and practices were 32.6 and 53.1, respectively. Additionally, a lack of thorough understanding of COVID-19 prevention, including proper mask-wearing techniques, adherence to physical distancing practices, avoiding sharing masks, etc., can contribute to transmitting the virus irrespective of mothers’ knowledge and practice level. We refrained from classifying the mothers’ scores as good, better, or poor, as this is primarily a theoretical consideration and may not provide an appropriate empirical basis for decision-making.**

7. Results and Discussions: Tables are not clearly understandable, particularly table 3. Tables should be more structured with all the required information.

**Response: Thank you for your suggestion on the tables. Each table contains specific notes at the end to clarify the values, which would help understand and interpret the table values. Nevertheless, we have included another note at the end of Table 3 for better clarity. (Page- 18)**

8. Limitation: This study only considered mothers from villages. Since mothers from urban areas were not considered, there is a concern with the generalization of the findings. Thus, it can be a limitation.

**Response: Thank you for your insight. We have stated that the findings cannot be generalized to different contexts. This statement also applies to rural and urban mothers due to their distinct and incomparable settings. (Page 22; line number- 334)**

9. Conclusion: Overall, the study recommendation needs to be aligned with the study objective. Therefore, it’s suggested to revise in line with the findings.

**Response: We have revised the recommendations to ensure they are closely aligned with the findings of the study. (Page- 22; Line number- 336)**

10. There are line and word spacing problems throughout the paper.

**Response: We are grateful for your observations on the formatting and spacing. We have thoroughly checked the manuscript and ensured that the revised version strictly complies with the PLOS Global Public Health submission guidelines.**

11. There are a few spelling mistakes and writing errors throughout the writing.

**Response: We value your feedback on the spelling and writing errors. We have carefully considered this and revised the manuscript accordingly.**
